# Epidemiologic patterns of human *Salmonella* serotype diversity in the USA, 1996–2016

**DOI:** 10.1017/S0950268819000724

**Published:** 2019-05-02

**Authors:** M. C. Judd, R. M. Hoekstra, B. E. Mahon, P. I. Fields, K. K. Wong

**Affiliations:** 1 Enteric Diseases Epidemiology Branch, Centers for Disease Control and Prevention, Atlanta, Georgia, USA; 2 Enteric Diseases Laboratory Branch, Centers for Disease Control and Prevention, Atlanta, Georgia, USA

**Keywords:** Food-borne infections, infectious disease epidemiology, *Salmonella*, salmonellosis

## Abstract

Although researchers have described numerous risk factors for salmonellosis and for infection with specific common serotypes, the drivers of *Salmonella* serotype diversity among human populations remain poorly understood. In this retrospective observational study, we partition records of serotyped non-typhoidal *Salmonella* isolates from human clinical specimens reported to CDC national surveillance by demographic, geographic and seasonal characteristics and adapt sample-based rarefaction methods from the field of community ecology to study how *Salmonella* serotype diversity varied within and among these populations in the USA during 1996–2016. We observed substantially higher serotype richness in children <2 years old than in older children and adults and steadily increasing richness with age among older adults. Whereas seasonal and regional variation in serotype diversity was highest among infants and young children, variation by specimen source was highest in adults. Our findings suggest that the risk for infection from uncommon serotypes is associated with host and environmental factors, particularly among infants, young children and older adults. These populations may have a higher proportion of illness acquired through environmental transmission pathways than published source attribution models estimate.

## Introduction

*Salmonella* is a diverse genus of zoonotic bacterial pathogens that cause an estimated 1.2 million human infections, 23 000 hospitalisations and 450 deaths annually in the USA [1]. Numerous reservoirs and the capacity for environmental persistence give *Salmonella* multiple entry points into the human population. Although *Salmonella* infections (salmonellosis) are thought to be primarily foodborne, estimates of the proportion of illness attributed to food, water and animal contact vary substantially or are based on sparse data [1–3], and no serotype-specific stratified estimates exist.

*Salmonella* serotypes may differ in their natural reservoirs, their geographic and seasonal distributions, and their ability to cause human infections [4–6]. Over 1300 serotypes have been isolated from cases of human disease and reported to the United States Centers for Disease Control and Prevention (CDC) since national surveillance began in 1963, and >2500 serotypes have been identified globally [7]. However, only a small proportion of these serotypes are regularly isolated from human clinical specimens [8]; 39 serotypes comprised >90% of isolates reported to the CDC in 2016 [9].

Although researchers have described numerous risk factors for salmonellosis and for infection with specific common serotypes, the drivers of *Salmonella* serotype diversity among human populations remain poorly understood. Traditional methods to describe salmonellosis risk factors and prominent reservoirs in the USA are limited in their ability to describe serotype diversity; case–control studies rely on the availability of substantial numbers of cases and are therefore not optimal for the characterisation of rare serotypes. They also often focus on outbreak-associated illness, which is thought to make up only a small fraction of all *Salmonella* cases and serotypes [10]. Environmental and animal sampling has limited usefulness in characterizing which reservoirs are likely to contribute significant diversity among human infections [11]. Methods developed in the field of community ecology may provide tools that are more suitable for tackling the problem of understanding serotype diversity. Rarefaction is a statistical method with applications in community ecology and microbiology, among others, used to describe natural community structure and diversity through the estimation and comparison of the ‘species richness’, i.e. the number of species or subtypes present among populations of different sizes [12–15]. Application of these methods to the study of epidemiologic patterns of *Salmonella* serotype diversity in humans may lead to a better understanding of exposure pathways and permit more accurate estimation of sources.

In this study, we partition *Salmonella* isolates from human clinical specimens reported to CDC national surveillance by demographic, geographic and seasonal characteristics and adapt rarefaction methods to study how *Salmonella* serotype diversity varied within and among these populations in the USA during 1996–2016.

## Methods

### National *Salmonella* surveillance

State and regional public health laboratories (hereafter, ‘reporting partners’) serotype *Salmonella* isolates cultured from human specimens according to the White–Kauffmann–Le Minor scheme [16] or forward difficult or unusual isolates to the CDC's National *Salmonella* Reference Laboratory for serotyping. Reporting partners then submit electronic records of these isolates to the CDC's Laboratory-based Enteric Disease Surveillance (LEDS) system. LEDS has been described in detail elsewhere [17]. In brief, cases of *Salmonella* infection are reported to LEDS with information on the serotype and specimen source of the isolate, date of specimen collection and basic demographic and geographic characteristics of the patient. Only the first isolation from the most invasive specimen source (e.g. blood *vs.* stool) within a 30-day period for each infection is counted.

### Study data

Cases from LEDS were included for analysis if (1) the isolate was cultured from a specimen collected during 1996–2016, (2) the isolate was fully serotyped, (3) the reporting partner serotyped an annual median of ⩾75% of their isolates during the study period, (4) the serotype was reported to LEDS at least twice in each year that it was reported by reporting partners satisfying the third criterion, and (5) the serotype was not Typhi, Paratyphi A, Paratyphi B or Paratyphi C. We applied the third criterion to reduce reporting bias, the fourth criterion to reduce random error and ensure that each serotype was circulating during the study period, and the fifth criterion to reduce the cases commonly associated with international travel.

We analysed the cases by patient age, sex, source of clinical specimen, geographic region and season. We calculated the age for patients with a known date of birth using the date of specimen collection and used the reported age for patients with missing date of birth. We categorised specimen source (blood, stool, urine, other or unknown), geographic region of infection (see https://www2.census.gov/geo/pdfs/maps-data/maps/reference/us_regdiv.pdf for state listing) and season using date of specimen collection (Winter, December 15–March 14; Spring, March 15–June 14; Summer, June 15–September 14; and Fall, September 15–December 14). We categorised the serotypes based on the median number of annual cases as very rare (2–9), rare (10–99), common (100–999) and very common (⩾1000). Cases without age data and sex data were excluded from rarefaction analyses involving age and sex. Cases with unknown patient state of residence were assumed to have occurred in the region they were reported. Cases without specimen source data, with specimen sources categorised as ‘Other’, or for which isolates were cultured from both stool and urine were excluded for rarefaction analyses involving specimen source to reduce the likelihood of misclassifying a stool-contaminated urine specimen as representing a true urinary tract infection.

### Serotype diversity analyses

We defined *Salmonella* serotype richness as the number of different *Salmonella* serotypes causing human infection in the USA during 1996–2016. We used sample-based rarefaction methods [18–20] to describe the epidemiologic patterns of serotype diversity by aggregating cases into temporally and geographically defined samples (i.e. serotype-specific case counts for each combination of state, season and year) and estimating serotype richness and 95% confidence intervals at the largest common number of cases among all categories in a given analysis. We performed univariate analyses to estimate and compare serotype richness by age group, sex, specimen source, geographic region and season and bivariate analyses to describe serotype richness variation within age groups by sex, specimen source, geographic region and season. We validated our richness comparisons by plotting rarefaction curves of the estimated serotype richness against the number of cases for each category and visually confirmed that the curves did not intersect at case counts greater than the largest common number (i.e. the category-specific richness rank order did not change) [21]. We defined the referent category as the category with the fewest cases and we assumed that the serotypes in the referent category were a subset of the serotypes in the largest category. Differences in serotype richness among categories with non-overlapping confidence intervals were deemed statistically significant. We adjusted all analyses for natural heterogeneity in spatial and temporal serotype frequency distributions. Sample-based rarefaction was performed using the EstimateS software package (version 9.1.0, Windows) [22]. All figures were produced using the ggplot2 package (version 2.2.1) [23] for R 3.4.2.

## Results

### Study population

Of the 815 789 cases and 1191 serotypes reported to LEDS during 1996–2016, we excluded 77 783 (9.5%) cases with unavailable or incomplete serotype data, 31 927 (3.9%) cases and 13 (1.1%) serotypes from five reporting partners who fully serotyped an annual median of <75% of their isolates during the study period (Florida, Montana, Nebraska, Texas and Wyoming), 2751 (0.3%) cases and 556 (47.0%) serotypes from reporting partners reporting the serotype only once in a given year, and 12 849 (1.6%) cases and four (0.3%) typhoidal serotypes. After exclusions, our study population consisted of 690 479 cases and 618 serotypes. We categorised 516 serotypes as very rare, 68 as rare, 29 as common and five as very common (Supplementary Table S1, available on the Cambridge Core website).

Of the 690 479 cases and 618 serotypes in our study population, we excluded 57 651 (8.3%) cases lacking age data and the four (0.7%) serotypes unique to them for analyses involving age and 44 859 (6.5%) cases lacking sex data and the nine (1.5%) serotypes unique to them for analyses involving sex. For analyses involving specimen source, we excluded 48 145 (7.0%) cases lacking specimen source data and the five (0.8%) serotypes unique to them, 3637 (0.5%) cases with isolates from specimen sources categorised as ‘Other’ and three (0.5%) serotypes unique to them, and 396 (<0.1%) cases for which isolates came from both stool and urine. No reports lacked geographic data or date of specimen collection. Case and serotype distributions by variables of interest were not meaningfully affected by the exclusion process. Very rare and rare serotypes comprised nearly 95% of all serotypes but <10% of all cases.

### Age

**Fig. 1.**
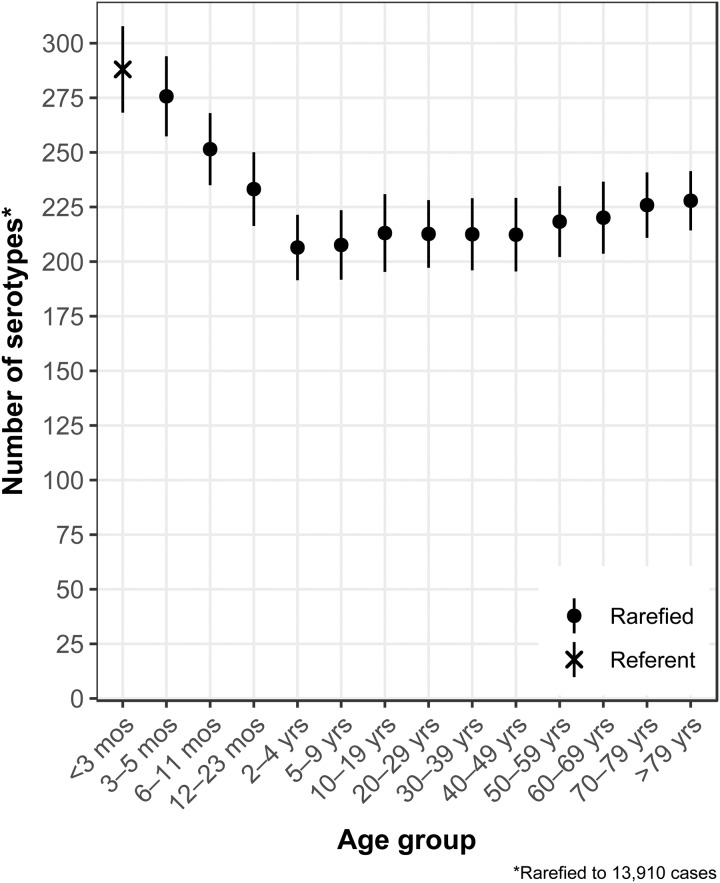
*Salmonella* serotype richness by age group, USA, 1996–2016.

The median (1st–3rd quartiles) age was 25.6 (4.7–50.9) years. An average of 51.7% of all serotypes identified during the study period was reported in every age group. Infants <3 months old had the highest proportion of cases caused by very rare and rare serotypes (4.4% and 14.8%, respectively; Table 1), followed by infants 3–5 months old (4.0% and 14.5%; Table 1). Very rare and rare serotypes also contributed >10% of cases among infants 6–11 months old, children 12–23 months old and adults >70 years old (Table 1). When case counts in each age group were rarefied (i.e. standardised using rarefaction methods) to the number observed in infants <3 months old, serotype richness was highest in infants and lowest among children 2–9 years old (Fig. 1). Richness increased after age 10 but never reached the levels observed in the very young.

**Table 1.** Percentage of *Salmonella* isolates reported to the CDC Laboratory-based Enteric Disease Surveillance (LEDS) system, by serotype rareness^a^ and demographic, geographic and temporal characteristics, USA, 1996–2016

Population	Very rare	Rare	Common	Very common
Age group				
<3 months	4.4	14.8	33.5	47.3
3–5 months	4.0	14.5	34.8	46.7
6–11 months	3.0	11.1	34.5	51.4
12–23 months	2.3	8.6	33.2	55.9
2–4 years	1.6	5.7	29.8	63.0
5–9 years	1.4	5.1	28.5	64.9
10–19 years	1.5	5.4	29.5	63.6
20–29 years	1.5	6.1	30.9	61.5
30–39 years	1.5	6.3	31.5	60.7
40–49 years	1.6	6.6	30.8	61.0
50–59 years	1.8	7.1	31.0	60.1
60–69 years	1.9	7.9	31.2	58.9
70–79 years	2.1	9.4	32.4	56.2
>79 years	2.2	10.2	33.5	54.1
Sex				
Female	1.9	7.4	31.8	58.8
Male	1.8	7.3	30.5	60.4
Season^b^				
Spring	2.2	7.8	32.4	57.6
Summer	1.6	6.7	31.3	60.5
Fall	1.7	7.3	30.5	60.5
Winter	2.4	8.9	29.3	59.3
Census region				
Midwest	1.7	7.2	33.0	58.1
Northeast	2.1	7.5	28.8	61.7
South	1.6	6.0	29.1	63.3
West	2.4	9.5	34.1	54.0
Specimen source				
Blood	2.7	12.2	27.2	57.8
Stool	1.7	6.7	30.7	61.0
Urine	3.7	13.0	41.2	42.1

a We categorised serotypes based on median number of annual cases as very rare (2–9), rare (10–99), common (100–999) and very common (⩾1000).

b Winter, December 15–March 14; Spring, March 15–June 14; Summer, June 15–September 14; and Fall, September 15–December 14.

### Sex

Although nearly all serotypes were identified in both sexes, there were more cases and serotypes among females than males (Table 2). Males had more infections with very common serotypes than females (Table 1). When case counts were rarefied to the number observed in males, we observed no significant variation in serotype richness between males and females, overall or by age group.

**Table 2.** Unadjusted counts of *Salmonella* cases and serotypes reported to the CDC Laboratory-based Enteric Disease Surveillance (LEDS) system, by demographic, geographic and temporal characteristics, USA, 1996–2016

Population	Cases		Serotypes^a^	
	*n*	%	*n*	%
Age group^b^				
<3 months	13 910	2.2	288	46.9
3–5 months	18 334	2.9	303	49.3
6–11 months	27 352	4.3	310	50.5
12–23 months	35 246	5.6	312	50.8
2–4 years	66 279	10.5	335	54.6
5–9 years	52 436	8.3	324	52.8
10–19 years	62 757	9.9	344	56.0
20–29 years	69 315	11.0	354	57.7
30–39 years	61 455	9.7	348	56.7
40–49 years	61 549	9.7	337	54.9
50–59 years	58 926	9.3	333	54.2
60–69 years	46 745	7.4	318	51.8
70–79 years	34 869	5.5	301	49.0
>79 years	23 655	3.7	266	43.3
Total	632 828		614	
Sex^c^				
Female	338 691	52.5	558	91.6
Male	306 929	47.5	540	88.7
Total	645 620		609	
Season^d^				
Spring	150 843	21.8	472	76.4
Summer	266 234	38.6	494	79.9
Fall	173 584	25.1	460	74.4
Winter	99 818	14.5	450	72.8
Total	690 479		618	
Census region				
Midwest	144 037	20.9	397	64.2
Northeast	152 067	22.0	424	68.6
South	231 628	33.5	466	75.4
West	162 747	23.6	425	68.8
Total	690 479		618	
Specimen source^e^				
Blood	33 565	5.3	320	52.5
Stool	568 534	89.1	591	96.9
Urine	36 202	5.7	374	61.3
Total	638 301		610	

a *n*: Count of different serotypes; %: Percentage of total different serotypes.

b In total, 57 651 cases with unknown age were excluded.

c In total, 44 859 cases with unknown sex were excluded.

d Winter, December 15–March 14; Spring, March 15–June 14; Summer, June 15–September 14; and Fall, September 15–December 14.

e In total, 396 cases with isolates from stool and urine, 3637 cases with other specimen source and 48 145 cases with unknown specimen source were excluded.

### Season

**Fig. 2.**
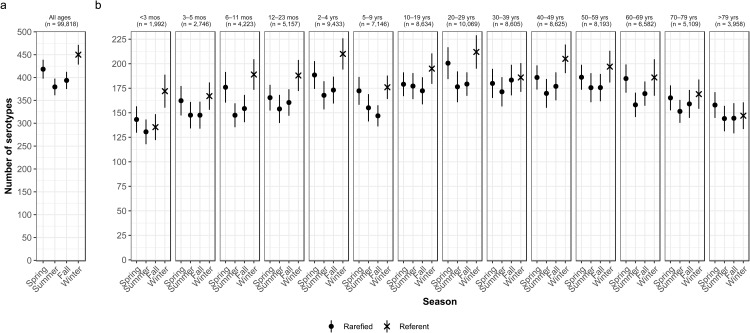
Seasonal variation of *Salmonella* serotype richness, (a) overall and (b) by age group, USA, 1996–2016.

Overall case counts peaked in summer (38.6%) and were lowest in winter (14.5%) (Table 2). However, very rare and rare serotypes contributed >10% of all cases in winter (Table 1), and overall serotype richness peaked in winter (Fig. 2a) when case counts were rarefied to the number occurring during winter.

### Season by age

We observed higher serotype richness in winter than in summer in most age groups, with significant variations among most children <5 years old (infants <3 months and 6–11 months, and children 12–23 months old and 2–4 years old) and among adults 20–29 and 40–49 years old (Fig. 2b). Infants <3 months old had the same adjusted case counts in Fall and Winter (Fig. 2b). Children 2–4 years old showed the greatest significant seasonal variation in serotype richness (210 serotypes in winter *vs.* 168 in summer) (Fig. 2b).

### Census region

**Fig. 3.**
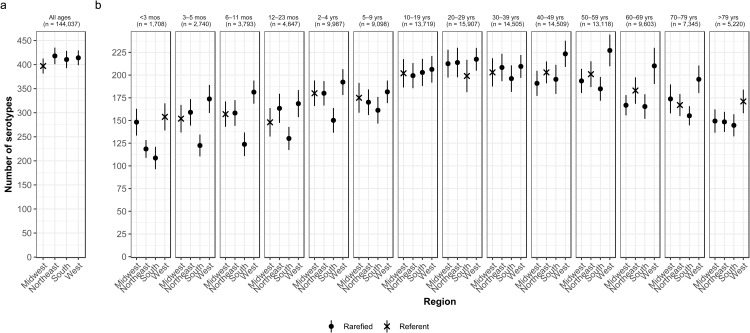
Geographic variation of *Salmonella* serotype richness, (a) overall and (b) by age group, USA, 1996–2016.

Whereas the Midwest, Northeast and West contributed roughly the same percentages of cases (20.9–23.6%), the South contributed the most (33.5%; Table 2). Between 64.2% and 75.4% of all serotypes were observed in each census region (Table 2). Very rare and rare serotypes contributed >10% of cases in the West (Table 1). The Northeast had the highest serotype richness and the Midwest had the lowest when case counts were rarefied to the number reported in the Midwest, but these differences were not significant by geographic region alone (Fig. 3a).

### Census region by age

We observed that the South had the lowest serotype richness and the West had the highest serotype richness for most age groups, with significant geographic variation among children <5 years old and adults ⩾50 years old (Fig. 3b). Infants 6–11 months old showed the largest significant variation, with higher serotype richness in the West than in the South (181 *vs.* 124 serotypes) (Fig. 3b).

### Specimen source

**Fig. 4.**
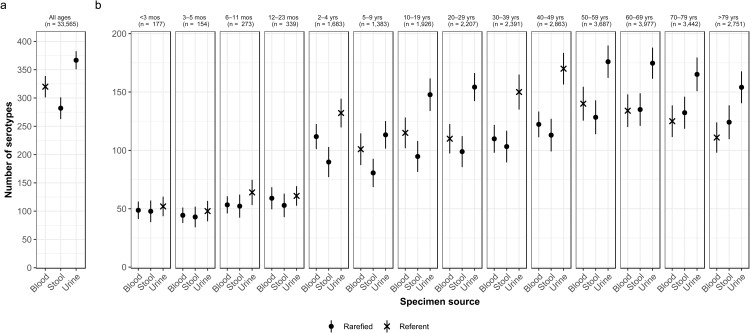
Specimen source variation of *Salmonella* serotype richness, (a) overall and (b) by age group, USA 1996–2016.

The majority of cases and serotypes were identified from stool (Table 2). Very rare and rare serotypes contributed >10% of cases in which *Salmonella* was isolated from blood or urine (Table 1). Although a minority of all cases were identified from urine and blood, 61.3% and 52.5% of all serotypes were identified from these specimen sources, respectively (Table 2). Cases identified from urine specimens were more often attributed to very rare and rare serotypes (16.7% of cases) compared with other specimen sources (blood, 14.9%; stool, 8.4%). We observed significantly higher serotype richness in urine than in blood or stool when case counts were rarefied to the number reported from blood (Fig. 4a).

### Specimen source by age

We consistently observed higher serotype richness in urine than in blood or stool in all age groups, with significant differences among individuals 2–4 and ⩾10 years old (Fig. 4b). Adults 40–49 years old showed the largest significant difference, with higher serotype richness in isolates from urine than from blood or stool (170 *vs.* 122 and 113 serotypes, respectively) (Fig. 4b).

## Discussion

Substantial demographic, geographic and temporal variations in *Salmonella* serotype diversity suggest that host and environmental factors contribute to the risk of infection from uncommon serotypes.

We observed substantially higher serotype richness in children <2 years old than in older children and adults and steadily increasing richness in older adults. We hypothesise that a combination of host and environmental factors drives higher serotype diversity in infants and young children whereas host factors may be the primary driver in older age groups.

Host risk factors such as an underdeveloped or senescing immune system and weak intestinal response to bacteria are important to pathophysiology early and late in life [24–26] and may allow serotypes that are less likely to cause illness in a healthy person to cause infection. Our study indicates that certain serotypes that are rarely isolated from adults are more commonly isolated from infants [27], and reports of outbreaks among infants indicate that such infections can be caused by markedly low doses [28].

In addition to the role of host factors, studies on the exposures of young children suggest that eating food plays a lesser role in the risk for salmonellosis than it does in older age groups. Contamination of the child's home with diverse environmental sources of *Salmonella* introduced by household members [29, 30] and by pets, especially reptiles [31], coupled with exploratory touching and tasting behaviours characteristic of infancy has been identified as an important transmission pathway. Exposure to environmental sources harbouring high serotype diversity (particularly surface waters [32]) and differing serotype profiles than food-related sources of infection [33] may also play an important role. However, we cannot discount the effect of higher healthcare-seeking rates for young children [34] on high serotype diversity since the chance of isolating an additional serotype, and thus increasing the measured richness, increases disproportionately in subgroups like infants in which a larger proportion of all true infections are detected.

We observed more seasonal and regional variation in serotype richness among infants and young children than in older age groups. This provides additional evidence for the importance of serotypically diverse environmental exposures in driving serotype diversity among infants and young children. Ambient environmental factors, such as seasonal precipitation, temperature and proximity to common animal reservoirs or agricultural activity may favour the presence and persistence of certain serotypes in environments commonly encountered by humans [33, 35]. Higher serotype richness in the winter despite fewer infections may reflect the impact of suboptimal growth conditions on the composition of *Salmonella* populations in the environment, leading to a decreased presence of common pathogenic serotypes [33] and a relatively increased presence of uncommon serotypes. It may also reflect higher bacterial detection during a period of higher healthcare-seeking rates due to other pathogens, such as viruses. Similarly, lower serotype richness in most age groups during summer may be due to environmental conditions favouring the persistence of common serotypes or more common exposure to less serotypically diverse sources associated with foodborne outbreaks, which peak in the summer. Reasons for higher serotype diversity among infants in the West than other geographic regions are less clear, but may reflect more serotypically diverse reservoirs or the decreased environmental presence of several common serotypes that cluster in the South [36].

Serotype diversity in age groups other than infants and young children exhibited less geographic and seasonal variation and more variation by specimen source. We observed higher richness among urine specimens from older children and adults compared with blood and stool. This difference was largest among young adults and smaller in older age groups. The reasons for this pattern are unknown, but it may be related to the elevated incidence of *Salmonella* infection identified from urine among adults and women of child-bearing age [37, 38]. However, this study does not distinguish between urinary tract infections and asymptomatic bacteriuria. Host risk factors, such as immunocompromising conditions, anatomic abnormalities, pregnancy status and sexual activity, may affect both the risk of urinary tract infection or bacteriuria and the diversity of serotypes that can be isolated from urine [39]. The excess serotypes identified in urine may represent those more likely to cause extraintestinal infections [40] or those less likely to cause gastrointestinal illness and only detected in urinary tract infections.

Our study has several limitations. The quality and quantity of passive surveillance data vary by reporting partner and over time, which may reflect the availability of resources for serotyping isolates and submitting electronic reports of infection. For every reported case of *Salmonella*, an estimated 28 cases go unreported [1]. Therefore, our rarefaction models likely represent the most common serotypes for the demographic, geographic and temporal variables we analysed, but may underestimate the number of rare serotypes, especially among populations less likely to seek care. This bias likely has minimal effect on our univariate analyses of age, season and specimen source due to their large sample sizes, but may be important for estimates in smaller sample size geographic analyses, especially among reporting partners whose volume of cases varies significantly over time. Geographic aggregation to census region may partly adjust for this bias, but to a lesser degree in the South than in other census regions since both Florida and Texas were excluded from all analyses. We could not adjust for differential case ascertainment by reporting catchment area based on active *vs.* passive surveillance. Ten of 52 (19%) LEDS reporting partners submit salmonellosis cases ascertained by active surveillance to the Foodborne Diseases Active Surveillance Network (FoodNet) operating in their jurisdictions; the remaining 44 conduct passive surveillance. This bias is likely minimal and non-differential for age, season and specimen source analyses; while active surveillance results in higher case ascertainment in the FoodNet catchment area than elsewhere, rates of serotype ascertainment are not meaningfully different. Finally, our estimates may not be fully representative of national serotype diversity because five reporting partners with <75% of isolates fully serotyped were excluded.

## Conclusions

Our novel approach to understanding the risk factors associated with salmonellosis finds that variations in serotype diversity may be primarily driven by host and environmental factors. Populations affected by a more diverse range of serotypes may have a higher proportion of environmentally-acquired illness than current source attribution models estimate. Efforts to refine the estimates of source attribution and to reduce the burden of salmonellosis should consider the contribution of environmental exposures and host factors and the effects of geography and seasonality, particularly among infants and young children. Population surveys and active enhanced surveillance can further explore the exposures in subpopulations based on the hypotheses raised by this analysis. Environmental analyses and additional study into host factors that may influence pathogen diversity, such as the host microbiome, can also be used to investigate diversity dynamics.

Further application of rarefaction methods to *Salmonella* epidemiology may provide new insights into exposure pathways by suggesting factors associated with exposure to serotypically diverse *Salmonella* reservoirs. Exploration of subtype diversity using molecular typing methods and replication of our study in other countries with similar sized populations is also warranted. Finally, broader application of rarefaction methods to the epidemiology of other enteric pathogens may also yield insights into their exposure pathways, suggest common underlying processes that drive diversity across pathogens and deepen our understanding of enteric disease epidemiology.

## Supplementary material

For supplementary material accompanying this paper visit https://doi.org/10.1017/S0950268819000724. click here to view supplementary material
