# Supplementary material for: Epidemiologic patterns of human Salmonella serotype diversity in the USA, 1996–2016
Source: Epidemiol Infect. 2019 May 2;147:e187. doi: 10.1017/S0950268819000724 (PMC6518743; doi:10.1017/S0950268819000724)
Supplement: Supplementary file 1 [file S0950268819000724sup001.docx]

*Epidemiology and Infection*

**Epidemiologic Patterns of Human *Salmonella* Serotype Diversity in the United States, 1996–2016**

M. C. JUDD, R. M. HOEKSTRA, B. E. MAHON, P. I. FIELDS, K. K. WONG

Supplementary Material

Supplementary Table S1. Serotypes isolated from human clinical specimens reported to the CDC Laboratory-based Enteric Disease Surveillance (LEDS) system, categorized by median annual reporting frequency, United States, 1996**–**2016^[[1]](#footnote-1)^.

| **Frequency category^[[2]](#footnote-2)^** | **Serotype^[[3]](#footnote-3)^** | **Median annual cases** |
| --- | --- | --- |
| Very common | Typhimurium | 6712 |
| Very common | Enteritidis | 6705 |
| Very common | Newport | 3706 |
| Very common | Javiana | 1705 |
| Very common | Heidelberg | 1576 |
| Common | I 4,[5],12:i:- | 940 |
| Common | Montevideo | 889 |
| Common | Muenchen | 818 |
| Common | Saintpaul | 688 |
| Common | Oranienburg | 676 |
| Common | Braenderup | 601 |
| Common | Infantis | 590 |
| Common | Thompson | 505 |
| Common | Mississippi | 449 |
| Common | Paratyphi B var. L(+) tartrate+ | 403 |
| Common | Agona | 398 |
| Common | Hadar | 286 |
| Common | Poona | 275 |
| Common | Berta | 257 |
| Common | Bareilly | 236 |
| Common | Anatum | 227 |
| Common | Stanley | 204 |
| Common | Hartford | 190 |
| Common | Mbandaka | 187 |
| Common | Schwarzengrund | 177 |
| Common | Litchfield | 175 |
| Common | I 13,23:b:- | 168.5 |
| Common | Panama | 161 |
| Common | Senftenberg | 142 |
| Common | Sandiego | 141 |
| Common | Derby | 131 |
| Common | Norwich | 117 |
| Common | Give | 109 |
| Common | I 4,[5],12:b:- | 105 |
| Common | Rubislaw | 100 |
| Rare | Miami | 94 |
| Rare | Dublin | 93 |
| Rare | Brandenburg | 87 |
| Rare | Weltevreden | 79 |
| Rare | Kentucky | 78 |
| Rare | Manhattan | 78 |
| Rare | Virchow | 76 |
| Rare | Gaminara | 74 |
| Rare | Reading | 74 |
| Rare | Adelaide | 71 |
| Rare | Muenster | 70 |
| Rare | Bovismorbificans | 69 |
| Rare | Uganda | 67 |
| Rare | Ohio | 64 |
| Rare | Blockley | 56 |
| Rare | Pomona | 56 |
| Rare | Hvittingfoss | 45 |
| Rare | Inverness | 43 |
| Rare | Urbana | 43 |
| Rare | Kiambu | 41 |
| Rare | Tennessee | 37 |
| Rare | Havana | 36 |
| Rare | Cerro | 35 |
| Rare | Johannesburg | 35 |
| Rare | Bredeney | 30 |
| Rare | London | 29 |
| Rare | Oslo | 29 |
| Rare | Worthington | 29 |
| Rare | Chester | 28 |
| Rare | Albany | 27 |
| Rare | Minnesota | 25 |
| Rare | I 6,7:-:1,5 | 24.5 |
| Rare | Telelkebir | 23 |
| Rare | Corvallis | 21.5 |
| Rare | Cubana | 21 |
| Rare | Cotham | 20.5 |
| Rare | Lomalinda | 18 |
| Rare | Ealing | 17 |
| Rare | I 6,7:k:- | 17 |
| Rare | Agbeni | 16 |
| Rare | Alachua | 16 |
| Rare | IV 48:g,z51:- | 16 |
| Rare | Baildon | 15 |
| Rare | Eastbourne | 15 |
| Rare | I 9,12:l,z28:- | 15 |
| Rare | II 13,23:b:[1,5]:z42 | 15 |
| Rare | IV 50:z4,z23:- | 15 |
| Rare | Meleagridis | 15 |
| Rare | Indiana | 13 |
| Rare | IV 50:g,z51:- | 13 |
| Rare | Monschaui | 13 |
| Rare | Nchanga | 12.5 |
| Rare | Choleraesuis | 12 |
| Rare | I 9,12:-:1,5 | 12 |
| Rare | IIIb 61:l,v:1,5,7 | 12 |
| Rare | Potsdam | 12 |
| Rare | I 3,10:-:l,w | 11.5 |
| Rare | IIIa 18:z4,z23:- | 11 |
| Rare | IIIa 41:z4,z23:- | 11 |
| Rare | Pensacola | 11 |
| Rare | Rissen | 11 |
| Rare | Carrau | 10.5 |
| Rare | I 6,8:e,h:- | 10.5 |
| Rare | I 4,[5],12:-:1,2 | 10 |
| Rare | I 4,[5],12:r:- | 10 |
| Rare | I 6,7:r:- | 10 |
| Rare | IV 44:z4,z23:- | 10 |
| Very rare | Ibadan | 9 |
| Very rare | Kintambo | 9 |
| Very rare | Kottbus | 9 |
| Very rare | Othmarschen | 9 |
| Very rare | Durban | 8.5 |
| Very rare | Irumu | 8.5 |
| Very rare | Altona | 8 |
| Very rare | Chailey | 8 |
| Very rare | I 6,8:d:- | 8 |
| Very rare | Itami | 8 |
| Very rare | Singapore | 8 |
| Very rare | IIIa 48:g,z51:- | 7.5 |
| Very rare | Madelia | 7.5 |
| Very rare | Arechavaleta | 7 |
| Very rare | Chittagong | 7 |
| Very rare | Edinburg | 7 |
| Very rare | I 6,8:-:1,2 | 7 |
| Very rare | IIIb 50:k:z | 7 |
| Very rare | IIIb 50:r:z | 7 |
| Very rare | IV 45:g,z51:- | 7 |
| Very rare | Livingstone | 7 |
| Very rare | Nima | 7 |
| Very rare | Saphra | 7 |
| Very rare | Stanleyville | 7 |
| Very rare | Liverpool | 6.5 |
| Very rare | Aberdeen | 6 |
| Very rare | Apapa | 6 |
| Very rare | Choleraesuis var. Kunzendorf | 6 |
| Very rare | Cullingworth | 6 |
| Very rare | Fluntern | 6 |
| Very rare | I 3,10:-:1,5 | 6 |
| Very rare | I 6,7:e,h:- | 6 |
| Very rare | IIIa 13,23:z4,z24:- | 6 |
| Very rare | IIIb 48:i:z | 6 |
| Very rare | IV 6,7:z4,z24:- | 6 |
| Very rare | Putten | 6 |
| Very rare | Richmond | 6 |
| Very rare | Abony | 5.5 |
| Very rare | I 4,[5],12:-:1,5 | 5.5 |
| Very rare | Kingabwa | 5.5 |
| Very rare | Aarhus | 5 |
| Very rare | Amager | 5 |
| Very rare | Amsterdam | 5 |
| Very rare | Barranquilla | 5 |
| Very rare | Bassadji | 5 |
| Very rare | Bonariensis | 5 |
| Very rare | Clackamas | 5 |
| Very rare | Colindale | 5 |
| Very rare | Daytona | 5 |
| Very rare | Emek | 5 |
| Very rare | Glostrup | 5 |
| Very rare | Haifa | 5 |
| Very rare | I 3,10:-:1,7 | 5 |
| Very rare | I 3,10:e,h:- | 5 |
| Very rare | I 4,[5],12:d:- | 5 |
| Very rare | IV 16:z4,z32:- | 5 |
| Very rare | Orion | 5 |
| Very rare | Poano | 5 |
| Very rare | Sangera | 5 |
| Very rare | Sundsvall | 5 |
| Very rare | Tallahassee | 5 |
| Very rare | Wandsworth | 5 |
| Very rare | Westhampton | 5 |
| Very rare | Ago | 4.5 |
| Very rare | Concord | 4.5 |
| Very rare | Entebbe | 4.5 |
| Very rare | Florida | 4.5 |
| Very rare | I 9,12:a:- | 4.5 |
| Very rare | Napoli | 4.5 |
| Very rare | Taksony | 4.5 |
| Very rare | Abaetetuba | 4 |
| Very rare | Africana | 4 |
| Very rare | Anecho | 4 |
| Very rare | Assinie | 4 |
| Very rare | Bochum | 4 |
| Very rare | California | 4 |
| Very rare | Carmel | 4 |
| Very rare | Chandans | 4 |
| Very rare | Chichiri | 4 |
| Very rare | Falkensee | 4 |
| Very rare | Farsta | 4 |
| Very rare | Fayed | 4 |
| Very rare | Freetown | 4 |
| Very rare | Fresno | 4 |
| Very rare | I 4,[5],12:e,h:- | 4 |
| Very rare | I 6,7:-:1,6 | 4 |
| Very rare | I 6,7:y:- | 4 |
| Very rare | I 9,12:i:- | 4 |
| Very rare | II 47:b:1,5 | 4 |
| Very rare | IIIa 44:z4,z23:- | 4 |
| Very rare | IIIa 48:z4,z24:- | 4 |
| Very rare | IIIa 53:z4,z23:- | 4 |
| Very rare | IIIb 60:r:e,n,x,z15 | 4 |
| Very rare | IIIb 61:k:1,5,[7] | 4 |
| Very rare | IIIb 61:z52:z53 | 4 |
| Very rare | Isangi | 4 |
| Very rare | Israel | 4 |
| Very rare | IV 43:z4,z23:- | 4 |
| Very rare | IV 44:z36,[z38]:- | 4 |
| Very rare | IV 44:z4,z32:- | 4 |
| Very rare | Jangwani | 4 |
| Very rare | Kotu | 4 |
| Very rare | Lomita | 4 |
| Very rare | Luciana | 4 |
| Very rare | Maumee | 4 |
| Very rare | Michigan | 4 |
| Very rare | Mikawasima | 4 |
| Very rare | Milwaukee | 4 |
| Very rare | Moscow | 4 |
| Very rare | Nottingham | 4 |
| Very rare | Romanby | 4 |
| Very rare | Soerenga | 4 |
| Very rare | Somone | 4 |
| Very rare | Takoradi | 4 |
| Very rare | Uppsala | 4 |
| Very rare | Washington | 4 |
| Very rare | Wentworth | 4 |
| Very rare | Aequatoria | 3.5 |
| Very rare | Farmsen | 3.5 |
| Very rare | I 6,7:-:1,2 | 3.5 |
| Very rare | II 58:l,z13,z28:z6 | 3.5 |
| Very rare | II 9,12:g,m,[s],t:[1,5,7]:[z42] | 3.5 |
| Very rare | IIIb 60:r:z | 3.5 |
| Very rare | IIIb 61:i:z53 | 3.5 |
| Very rare | Larochelle | 3.5 |
| Very rare | Lexington | 3.5 |
| Very rare | Newmexico | 3.5 |
| Very rare | Okatie | 3.5 |
| Very rare | Ruiru | 3.5 |
| Very rare | Sinstorf | 3.5 |
| Very rare | Vejle | 3.5 |
| Very rare | Waycross | 3.5 |
| Very rare | Widemarsh | 3.5 |
| Very rare | Aba | 3 |
| Very rare | Agoueve | 3 |
| Very rare | Alabama | 3 |
| Very rare | Alagbon | 3 |
| Very rare | Albert | 3 |
| Very rare | Aqua | 3 |
| Very rare | Australia | 3 |
| Very rare | Beaudesert | 3 |
| Very rare | Benin | 3 |
| Very rare | Birkenhead | 3 |
| Very rare | Blegdam | 3 |
| Very rare | Brezany | 3 |
| Very rare | Brive | 3 |
| Very rare | Bron | 3 |
| Very rare | Bsilla | 3 |
| Very rare | Cannstatt | 3 |
| Very rare | Coeln | 3 |
| Very rare | Denver | 3 |
| Very rare | Diguel | 3 |
| Very rare | Duisburg | 3 |
| Very rare | Durham | 3 |
| Very rare | Elomrane | 3 |
| Very rare | Fischerstrasse | 3 |
| Very rare | Gatuni | 3 |
| Very rare | Grumpensis | 3 |
| Very rare | Guinea | 3 |
| Very rare | Hindmarsh | 3 |
| Very rare | Holcomb | 3 |
| Very rare | Hull | 3 |
| Very rare | I 13,22:-:1,6 | 3 |
| Very rare | I 13,23:z:- | 3 |
| Very rare | I 16:d:- | 3 |
| Very rare | I 28:i:- | 3 |
| Very rare | I 3,10:-:1,6 | 3 |
| Very rare | I 4,[5],12:-:e,n,z15 | 3 |
| Very rare | I 4,[5],12:l,v:- | 3 |
| Very rare | I 43:k:- | 3 |
| Very rare | I 47:z4,z23:- | 3 |
| Very rare | I 6,7:b:- | 3 |
| Very rare | I 6,7:c:- | 3 |
| Very rare | I 6,7:l,w:- | 3 |
| Very rare | I 9,12:e,h:- | 3 |
| Very rare | I 9,12:g,z51:- | 3 |
| Very rare | I 9,12:l,v:- | 3 |
| Very rare | Idikan | 3 |
| Very rare | II 13,22:z29:1,5 | 3 |
| Very rare | II 21:b:1,5 | 3 |
| Very rare | II 4,12:l,w:e,n,x | 3 |
| Very rare | II 47:d:z39 | 3 |
| Very rare | IIIa 13,23:g,z51:- | 3 |
| Very rare | IIIa 43:z4,z23:- | 3 |
| Very rare | IIIa 48:z29:- | 3 |
| Very rare | IIIa 48:z4,z23:- | 3 |
| Very rare | IIIa 51:z4,z23:- | 3 |
| Very rare | IIIa 53:z4,z23,z32:- | 3 |
| Very rare | IIIa 56:z4,z23:- | 3 |
| Very rare | IIIb 16:z10:e,n,x,z15 | 3 |
| Very rare | IIIb 18:l,v:z | 3 |
| Very rare | IIIb 35:l,v:z35 | 3 |
| Very rare | IIIb 47:k:z35 | 3 |
| Very rare | IIIb 61:l,v:z | 3 |
| Very rare | IIIb 61:r:z | 3 |
| Very rare | Ilugun | 3 |
| Very rare | IV 11:z4,z23:- | 3 |
| Very rare | IV 40:z4,z23:- | 3 |
| Very rare | IV 44:z4,z24:- | 3 |
| Very rare | IV 48:z4,z32:- | 3 |
| Very rare | Kande | 3 |
| Very rare | Kedougou | 3 |
| Very rare | Koketime | 3 |
| Very rare | Lagos | 3 |
| Very rare | Lattenkamp | 3 |
| Very rare | Lika | 3 |
| Very rare | Lindenburg | 3 |
| Very rare | Maricopa | 3 |
| Very rare | Matadi | 3 |
| Very rare | Matopeni | 3 |
| Very rare | Ndolo | 3 |
| Very rare | Newyork | 3 |
| Very rare | Orientalis | 3 |
| Very rare | Ouakam | 3 |
| Very rare | Papuana | 3 |
| Very rare | Raus | 3 |
| Very rare | Roodepoort | 3 |
| Very rare | Saarbruecken | 3 |
| Very rare | Saugus | 3 |
| Very rare | Shubra | 3 |
| Very rare | Stockholm | 3 |
| Very rare | Telhashomer | 3 |
| Very rare | Toucra | 3 |
| Very rare | Tsevie | 3 |
| Very rare | Umbilo | 3 |
| Very rare | Uzaramo | 3 |
| Very rare | Wernigerode | 3 |
| Very rare | Yoruba | 3 |
| Very rare | Antsalova | 2.5 |
| Very rare | Ball | 2.5 |
| Very rare | Belem | 2.5 |
| Very rare | Bere | 2.5 |
| Very rare | Bousso | 2.5 |
| Very rare | Brazzaville | 2.5 |
| Very rare | Bukavu | 2.5 |
| Very rare | Caracas | 2.5 |
| Very rare | Hiduddify | 2.5 |
| Very rare | Horsham | 2.5 |
| Very rare | I 13,23:-:1,5 | 2.5 |
| Very rare | I 3,10:l,z13:- | 2.5 |
| Very rare | I 6,7:-:e,n,x | 2.5 |
| Very rare | I 6,7:d:- | 2.5 |
| Very rare | I 6,8:i:- | 2.5 |
| Very rare | I 6,8:z10:- | 2.5 |
| Very rare | II 50:b:z6 | 2.5 |
| Very rare | IIIa 41:z4,z32:- | 2.5 |
| Very rare | IIIa 47:z4,z23:- | 2.5 |
| Very rare | IIIa 50:z4,z23:- | 2.5 |
| Very rare | IIIb 35:k:e,n,x,z15 | 2.5 |
| Very rare | IIIb 38:(k):z35 | 2.5 |
| Very rare | IIIb 42:(k):z35 | 2.5 |
| Very rare | IIIb 47:k:- | 2.5 |
| Very rare | IIIb 47:r:z53 | 2.5 |
| Very rare | IIIb 48:r:z | 2.5 |
| Very rare | IIIb 48:z52:z | 2.5 |
| Very rare | IIIb 50:z:z52 | 2.5 |
| Very rare | IIIb 53:z10:z35 | 2.5 |
| Very rare | IIIb 53:z52:z53 | 2.5 |
| Very rare | IIIb 61:-:1,5,[7] | 2.5 |
| Very rare | Ituri | 2.5 |
| Very rare | IV 50:z4,z32:- | 2.5 |
| Very rare | Jamaica | 2.5 |
| Very rare | Kaduna | 2.5 |
| Very rare | Kisarawe | 2.5 |
| Very rare | Lansing | 2.5 |
| Very rare | Limete | 2.5 |
| Very rare | Lome | 2.5 |
| Very rare | Mendoza | 2.5 |
| Very rare | Nessziona | 2.5 |
| Very rare | Nigeria | 2.5 |
| Very rare | Nitra | 2.5 |
| Very rare | Oakland | 2.5 |
| Very rare | Obogu | 2.5 |
| Very rare | Oritamerin | 2.5 |
| Very rare | Overschie | 2.5 |
| Very rare | Praha | 2.5 |
| Very rare | Ridge | 2.5 |
| Very rare | Sanjuan | 2.5 |
| Very rare | Suelldorf | 2.5 |
| Very rare | Victoria | 2.5 |
| Very rare | Wangata | 2.5 |
| Very rare | Abadina | 2 |
| Very rare | Abortusequi | 2 |
| Very rare | Agama | 2 |
| Very rare | Ahuza | 2 |
| Very rare | Ajiobo | 2 |
| Very rare | Allandale | 2 |
| Very rare | Amoutive | 2 |
| Very rare | Anfo | 2 |
| Very rare | Angoda | 2 |
| Very rare | Ank | 2 |
| Very rare | Apeyeme | 2 |
| Very rare | Assen | 2 |
| Very rare | Babelsberg | 2 |
| Very rare | Bahrenfeld | 2 |
| Very rare | Banana | 2 |
| Very rare | Banco | 2 |
| Very rare | Bergen | 2 |
| Very rare | Bijlmer | 2 |
| Very rare | Bispebjerg | 2 |
| Very rare | *S. bongori* ser. 48:z35:- | 2 |
| Very rare | *S. bongori* ser. 48:z81:- | 2 |
| Very rare | Bonn | 2 |
| Very rare | Bournemouth | 2 |
| Very rare | Bradford | 2 |
| Very rare | Brancaster | 2 |
| Very rare | Brazil | 2 |
| Very rare | Brazos | 2 |
| Very rare | Bronx | 2 |
| Very rare | Brunei | 2 |
| Very rare | Buzu | 2 |
| Very rare | Canada | 2 |
| Very rare | Chicago | 2 |
| Very rare | Chincol | 2 |
| Very rare | Choleraesuis var. Decatur | 2 |
| Very rare | Colorado | 2 |
| Very rare | Cremieu | 2 |
| Very rare | Curacao | 2 |
| Very rare | Dahra | 2 |
| Very rare | Dakota | 2 |
| Very rare | Djugu | 2 |
| Very rare | Doel | 2 |
| Very rare | Eberswalde | 2 |
| Very rare | Ebrie | 2 |
| Very rare | Eppendorf | 2 |
| Very rare | Escanaba | 2 |
| Very rare | Essen | 2 |
| Very rare | Fomeco | 2 |
| Very rare | Friedenau | 2 |
| Very rare | Frintrop | 2 |
| Very rare | Fyris | 2 |
| Very rare | Galiema | 2 |
| Very rare | Galil | 2 |
| Very rare | Gambia | 2 |
| Very rare | Gatow | 2 |
| Very rare | Georgia | 2 |
| Very rare | Gera | 2 |
| Very rare | Glasgow | 2 |
| Very rare | Gloucester | 2 |
| Very rare | Gnesta | 2 |
| Very rare | Goettingen | 2 |
| Very rare | Goldcoast | 2 |
| Very rare | Guildford | 2 |
| Very rare | Hannover | 2 |
| Very rare | Hato | 2 |
| Very rare | Herston | 2 |
| Very rare | Hillingdon | 2 |
| Very rare | Hofit | 2 |
| Very rare | Homosassa | 2 |
| Very rare | I 11:-:e,n,x | 2 |
| Very rare | I 11:r:- | 2 |
| Very rare | I 13,22:b:- | 2 |
| Very rare | I 13,22:z:- | 2 |
| Very rare | I 16:b:- | 2 |
| Very rare | I 16:e,h:- | 2 |
| Very rare | I 16:l,v:- | 2 |
| Very rare | I 3,10:l,v:- | 2 |
| Very rare | I 3,10:r:- | 2 |
| Very rare | I 30:b:- | 2 |
| Very rare | I 38:k:- | 2 |
| Very rare | I 4,[5],12:-:1,7 | 2 |
| Very rare | I 40:-:e,n,x | 2 |
| Very rare | I 6,7:-:1,7 | 2 |
| Very rare | I 6,7:-:e,n,z15 | 2 |
| Very rare | I 6,7:a:- | 2 |
| Very rare | I 6,7:z10:- | 2 |
| Very rare | I 6,8:-:1,5 | 2 |
| Very rare | I 6,8:b:- | 2 |
| Very rare | I 6,8:l,v:- | 2 |
| Very rare | I 8,20:i:- | 2 |
| Very rare | I 9,12:undetermined | 2 |
| Very rare | II 16:m,t:- | 2 |
| Very rare | II 17:g,t:[e,n,x,z15] | 2 |
| Very rare | II 21:z10:[z6] | 2 |
| Very rare | II 47:b:e,n,x,z15 | 2 |
| Very rare | II 48:a:z6 | 2 |
| Very rare | II 48:d:z6 | 2 |
| Very rare | II 48:z39:z81 | 2 |
| Very rare | II 58:c:z6 | 2 |
| Very rare | II 6,7:-:1,6 | 2 |
| Very rare | II 9,12:z29:1,5 | 2 |
| Very rare | II 9,12:z39:1,7 | 2 |
| Very rare | IIIa 13,22:z4,z23:- | 2 |
| Very rare | IIIa 13,23:z4,z23,[z32]:- | 2 |
| Very rare | IIIa 18:z4,z32:- | 2 |
| Very rare | IIIa 21:g,z51:- | 2 |
| Very rare | IIIa 21:z29:- | 2 |
| Very rare | IIIa 21:z4,z23:- | 2 |
| Very rare | IIIa 35:z4,z23:- | 2 |
| Very rare | IIIa 35:z4,z32:- | 2 |
| Very rare | IIIa 40:g,z51:- | 2 |
| Very rare | IIIa 40:z4,z23:- | 2 |
| Very rare | IIIa 41:g,z51:- | 2 |
| Very rare | IIIa 41:z4,z24:- | 2 |
| Very rare | IIIa 42:g,z51:- | 2 |
| Very rare | IIIa 42:z4,z23:- | 2 |
| Very rare | IIIa 42:z4,z24:- | 2 |
| Very rare | IIIa 43:z29:- | 2 |
| Very rare | IIIa 44:z4,z24:- | 2 |
| Very rare | IIIa 47:g,z51:- | 2 |
| Very rare | IIIa 50:g,z51:- | 2 |
| Very rare | IIIa 51:g,z51:- | 2 |
| Very rare | IIIa 59:z4,z23:- | 2 |
| Very rare | IIIa 63:z4,z23:- | 2 |
| Very rare | IIIb 13,23:z:1,5 | 2 |
| Very rare | IIIb 38:(k):1,5,7 | 2 |
| Very rare | IIIb 38:l,v:z53 | 2 |
| Very rare | IIIb 48:c:z | 2 |
| Very rare | IIIb 48:z4,z24:- | 2 |
| Very rare | IIIb 50:k:- | 2 |
| Very rare | IIIb 50:k:z35 | 2 |
| Very rare | IIIb 50:k:z53 | 2 |
| Very rare | IIIb 50:l,v:z35 | 2 |
| Very rare | IIIb 50:r:1,5,(7) | 2 |
| Very rare | IIIb 50:z52:z35 | 2 |
| Very rare | IIIb 53:z10:z | 2 |
| Very rare | IIIb 60:k:z35 | 2 |
| Very rare | IIIb 60:z52:z | 2 |
| Very rare | IIIb 60:z52:z53 | 2 |
| Very rare | IIIb 61:c:z35 | 2 |
| Very rare | IIIb 61:i:z | 2 |
| Very rare | IIIb 61:l,v:z35 | 2 |
| Very rare | IIIb 61:r:z53 | 2 |
| Very rare | IIIb 65:(k):z | 2 |
| Very rare | IIIb 65:z10:e,n,x,z15 | 2 |
| Very rare | Inganda | 2 |
| Very rare | IV 21:z4,z23:- | 2 |
| Very rare | IV 40:z4,z24:- | 2 |
| Very rare | IV 40:z4,z32:- | 2 |
| Very rare | IV 43:z36,z38:- | 2 |
| Very rare | IV 43:z4,z32:- | 2 |
| Very rare | IV 48:z4,z23:- | 2 |
| Very rare | IV 6,7:z4,z23:- | 2 |
| Very rare | Joal | 2 |
| Very rare | Jos | 2 |
| Very rare | Jukestown | 2 |
| Very rare | Kaapstad | 2 |
| Very rare | Kalamu | 2 |
| Very rare | Kibi | 2 |
| Very rare | Kingston | 2 |
| Very rare | Kirkee | 2 |
| Very rare | Kisangani | 2 |
| Very rare | Kivu | 2 |
| Very rare | Kokomlemle | 2 |
| Very rare | Kouka | 2 |
| Very rare | Krefeld | 2 |
| Very rare | Kua | 2 |
| Very rare | Kumasi | 2 |
| Very rare | Landwasser | 2 |
| Very rare | Lawra | 2 |
| Very rare | Leeuwarden | 2 |
| Very rare | Lille | 2 |
| Very rare | Loanda | 2 |
| Very rare | Maastricht | 2 |
| Very rare | Malstatt | 2 |
| Very rare | Maracaibo | 2 |
| Very rare | Memphis | 2 |
| Very rare | Menston | 2 |
| Very rare | Mgulani | 2 |
| Very rare | Molade | 2 |
| Very rare | Mons | 2 |
| Very rare | Morehead | 2 |
| Very rare | Mowanjum | 2 |
| Very rare | Nagoya | 2 |
| Very rare | Newholland | 2 |
| Very rare | Ngili | 2 |
| Very rare | Ngor | 2 |
| Very rare | Offa | 2 |
| Very rare | Onderstepoort | 2 |
| Very rare | Onireke | 2 |
| Very rare | Oyonnax | 2 |
| Very rare | Pharr | 2 |
| Very rare | Plymouth | 2 |
| Very rare | Portland | 2 |
| Very rare | Quiniela | 2 |
| Very rare | Remo | 2 |
| Very rare | Riverside | 2 |
| Very rare | Rostock | 2 |
| Very rare | Sangalkam | 2 |
| Very rare | Sanktgeorg | 2 |
| Very rare | Sao | 2 |
| Very rare | Sendai | 2 |
| Very rare | Senegal | 2 |
| Very rare | Seremban | 2 |
| Very rare | Shipley | 2 |
| Very rare | Simi | 2 |
| Very rare | Spalentor | 2 |
| Very rare | Splott | 2 |
| Very rare | Stachus | 2 |
| Very rare | Tamberma | 2 |
| Very rare | Tampico | 2 |
| Very rare | Tanger | 2 |
| Very rare | Tarshyne | 2 |
| Very rare | Teddington | 2 |
| Very rare | Tees | 2 |
| Very rare | Teltow | 2 |
| Very rare | Texas | 2 |
| Very rare | Tilene | 2 |
| Very rare | Tokoin | 2 |
| Very rare | Tornow | 2 |
| Very rare | Travis | 2 |
| Very rare | Treforest | 2 |
| Very rare | Tripoli | 2 |
| Very rare | Tshiongwe | 2 |
| Very rare | Tucson | 2 |
| Very rare | Uccle | 2 |
| Very rare | Valdosta | 2 |
| Very rare | Vancouver | 2 |
| Very rare | Vanier | 2 |
| Very rare | Vilvoorde | 2 |
| Very rare | Waral | 2 |
| Very rare | Warnow | 2 |
| Very rare | Welikade | 2 |
| Very rare | Weslaco | 2 |
| Very rare | Wien | 2 |
| Very rare | Winston | 2 |
| Very rare | Wisbech | 2 |
| Very rare | Woodinville | 2 |
| Very rare | Yaba | 2 |
| Very rare | Zanzibar | 2 |
| Very rare | Zega | 2 |
| Very rare | Zerifin | 2 |

1. After exclusions, our study population consisted of 690,479 cases and 618 serotypes. [↑](#footnote-ref-1)
2. We categorized serotypes based on median number of annual cases as very rare (2–9) rare (10—99), common (100—999), and very common (≥1000) respectively [↑](#footnote-ref-2)
3. Serotype is a *Salmonella enterica* serotype unless otherwise specified. [↑](#footnote-ref-3)
